# High Temperature as a Risk Factor for Infectious Diarrhea in Shanghai, China

**DOI:** 10.2188/jea.JE20130012

**Published:** 2013-11-05

**Authors:** Xiaodan Zhou, Yanbing Zhou, Renjie Chen, Wenjuan Ma, Haiju Deng, Haidong Kan

**Affiliations:** 1 School of Public Health, Key Lab of Public Health Safety of the Ministry of Education, Fudan University, Shanghai, China; 2 Putuo District Center of Disease Control and Prevention, Shanghai, China

**Keywords:** temperature, diarrhea, time-series

## Abstract

**Background:**

Recent studies indicate that ambient temperature could be a risk factor for infectious diarrhea, but evidence for such a relation is limited in China.

**Methods:**

We investigated the short-term association between daily temperature and physician-diagnosed infectious diarrhea during 2008–2010 in Shanghai, China. We adopted a time-series approach to analyze the data and a quasi-Poisson regression model with a natural spline-smoothing function to adjust for long-term and seasonal trends, as well as other time-varying covariates.

**Results:**

There was a significant association between temperature and outpatient visits for diarrhea. A 1°C increase in the 6-day moving average of temperature was associated with a 2.68% (95% CI: 1.83%, 3.52%) increase in outpatient visits for diarrhea. We did not find a significant association between rainfall and infectious diarrhea.

**Conclusions:**

High temperature might be a risk factor for infectious diarrhea in Shanghai. Public health programs should focus on preventing diarrhea related to high temperature among city residents.

## INTRODUCTION

Infectious diarrhea is the largest contributor to the disease burden from poor water, sanitation, and hygiene.^1^ However, the risk factors for infectious diarrhea have not been fully identified. Using data on fluctuations in patient numbers and the survival characteristics of rotavirus, an important cause of diarrhea, previous studies showed that rotavirus infections are sensitive to seasonality.^2^^–^^4^ A study in Peru during the El Niño–Southern Oscillation found that daily admissions for diarrheal disease increased significantly when mean ambient temperature increased.^5^ In Bangladesh, the disease dynamics of cholera were associated with the El Niño–Southern Oscillation and regional temperature anomalies.^6^ Another study found that temperature, humidity, and rainfall might change the levels and ecology of pathogens, especially in marine environments.^7^ In places where rainwater is the source of domestic water and drinking water, lack of safe water supplies might contribute to diarrhea occurrence.^8^^–^^10^ Likewise, a sudden diarrhea outbreak might be driven by extremely heavy rainfall during a period of a few months or a season.^11^

Most previous studies were conducted in tropical countries or on islands. In China, the largest developing country in the world, infectious diarrhea causes significant mortality and morbidity^12^; however, few prior studies in the country have examined the association between temperature and infectious diarrhea.^13^^–^^16^ Moreover, most previous Chinese studies examined bacillary dysentery rather than infectious diarrhea. In this study, we examined the relationship between temperature and outpatient visits for diarrhea in Shanghai, the largest city in China.

## METHODS

### Data

Shanghai is located in eastern China. Neighboring the Yangtze River and the East China Sea, it has a total area of 6341 square kilometers.^17^ As a city with a subtropical humid monsoon climate, Shanghai has distinct seasons and a sufficient supply of fresh water and reaches its highest temperatures in July and August.

Putuo District Center Hospital is directly affiliated with the Shanghai Bureau of Health. Located in the northwestern part of Shanghai, it accepts patients from several nearby districts. The hospital has a special clinic for infectious diarrhea, which is responsible for screening patients with diarrhea symptoms. Briefly, medical history and blood samples of outpatients were collected upon arrival. Four common pathogens (including *Vibrio cholerae*, *Shigella dysenteriae*, salmonella, and *Vibrio parahaemolyticus*) were examined immediately. Only patients with a physician diagnosis of infectious diarrhea were accepted at this special clinic. The diagnostic criteria included symptoms reported at the first visit as well as pathogen examination. Patients with noninfectious diarrhea were treated at a regular clinic. We collected data on daily numbers of outpatient visits for infectious diarrhea from the special clinic, and the data collected from January 1, 2008 through December 31, 2010 were included in the present analysis.

Daily meteorologic data (including daily maximum temperature, daily mean temperature, average humidity, and rainfall) were obtained from the Shanghai Meteorological Bureau.

### Statistical analysis

Most infectious bacteria and viruses, such as *V cholerae*, dysentery bacillus, and rotavirus, spread through fecal–oral transmission. Healthy people can contract diarrhea after ingesting food or water contaminated by the feces of patients or carriers.^18^ With careful management of feces such transmission can be controlled, so that later cases will not be affected by early cases. Therefore, we assumed that the daily number of outpatients would have a Poisson distribution, for which independence of events is a necessity.

Because outpatient and weather data are linked by date, we used a time-series approach.^19^ A generalized additive model was used to adjust for long-term and seasonal trends, and for other time-varying covariates such as day of the week. We applied natural smooth functions of calendar time, with 7 degrees of freedom per year, to exclude unmeasured long-term and seasonal trends in the time-series dataset. We incorporated the natural smooth functions of relative humidity (3 degrees of freedom) to adjust for the potential nonlinear confounding effects of humidity. We also included day of the week and rainfall as indicator variables in the regression models. We then introduced mean temperature to estimate its association with infectious diarrhea.

Lag effects of mean temperature were also considered in building the models, because the temperature on the current day, as well as those on the preceding several days, could affect outpatient count on the current day.^20^ In addition to single-day lag models, a 6-day moving average of temperatures on the current day and previous 5 days (lag 05) was used to estimate the cumulative effect of temperature on infectious diarrhea.^21^

We conducted 2 sensitivity analyses. First, in addition to daily mean temperature, we examined the effects of daily maximum and minimum temperatures. Second, because it is not easy to determine the optimal degrees of freedom to control for long-term and seasonal trend in the models, we performed a sensitivity analysis to test the impact of degrees of freedom selection on the regression results. All analyses were conducted in R 2.15.1 using the mgcv package.

## RESULTS

From January 1, 2008 through December 31, 2010, we collected a total of 1096 days of daily data on weather conditions and outpatient visits for infectious diarrhea; 453 of the 1096 days were rainy. The total number of outpatient visits for infectious diarrhea was 27 270, among which 9841, 8091, and 9338 cases were recorded in 2008, 2009, and 2010, respectively.

Statistics on daily numbers of outpatient visit for infectious diarrhea, daily temperature (including minimum, maximum, and mean temperature), and relative humidity are summarized in Table 1. Over the 3 years studied, the number of daily outpatient visits ranged from 1 to 64 (median 23). The averages were 21.5°C for maximum temperature, 17.6°C for mean temperature, and 14.8°C for minimum temperature.

**Table 1.** Summary statistics of daily numbers of outpatient visit for infectious diarrhea, temperature, and relative humidity in Shanghai

	Min	P25	P50	P75	Max	Mean	SD
No. of outpatient visits for infectious diarrhea	1	17	23	30	64	24.7	9.90
Temperature (°C)							
Min	−5.9	7.0	15.5	22.7	32.1	14.8	8.97
Max	0.5	13.6	22.7	29.4	40.0	21.5	9.49
Mean	−3.0	9.9	18.7	25.4	35.4	17.6	9.02
Relative humidity (%)	24.0	62.0	71.0	80.0	98.0	70.4	13.73

Abbreviations: P25, first quartile; P50, median; P75, third quartile.

Table 2 shows year-specific data for various contributors to infectious diarrhea. Bacillary dysentery and typhoid and paratyphoid accounted for less than 0.5% of visits, and most patients affected by these illnesses were adults (Table 3). During the 3 years studied, only 1 child was diagnosed as having bacillary dysentery.

**Table 2.** Annual number of outpatient visit for infectious diarrhea, by cause, in Shanghai

	Bacillary dysentery	Typhoid and paratyphoid	Food poisoning	Other infectious diarrhea	Total
2008	29	0	15	9797	9841
2009	13	2	58	8018	8091
2010	30	2	0	9306	9338

**Table 3.** Annual number of outpatient visit for infectious diarrhea (excluding food poisoning), by age group, in Shanghai

	Children	Adults	Total
2008	108	9718	9826
2009	72	7961	8033
2010	37	9301	9338

**Figure 1.**
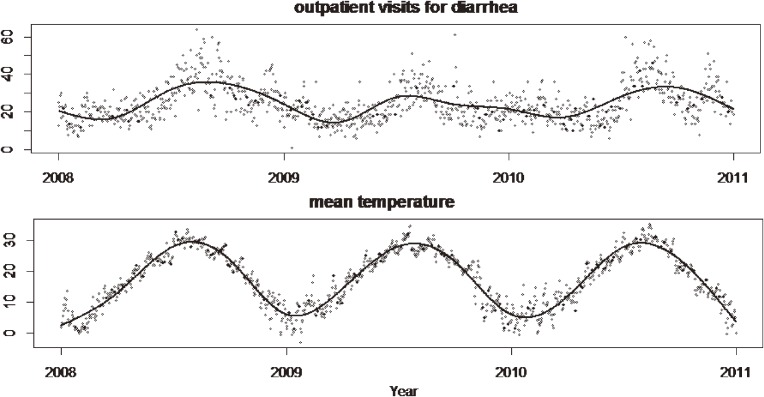
Seasonal trends in daily mean temperature and daily outpatient visits for diarrhea.

Figure 1 shows seasonal trends in daily mean temperature and number of daily outpatient visit for infectious diarrhea during 2008–2010. Both reached peaks in summer and decreased in winter. The 2 variables had similar trends, suggesting that temperature is positively associated with risk of infectious diarrhea.

**Figure 2.**
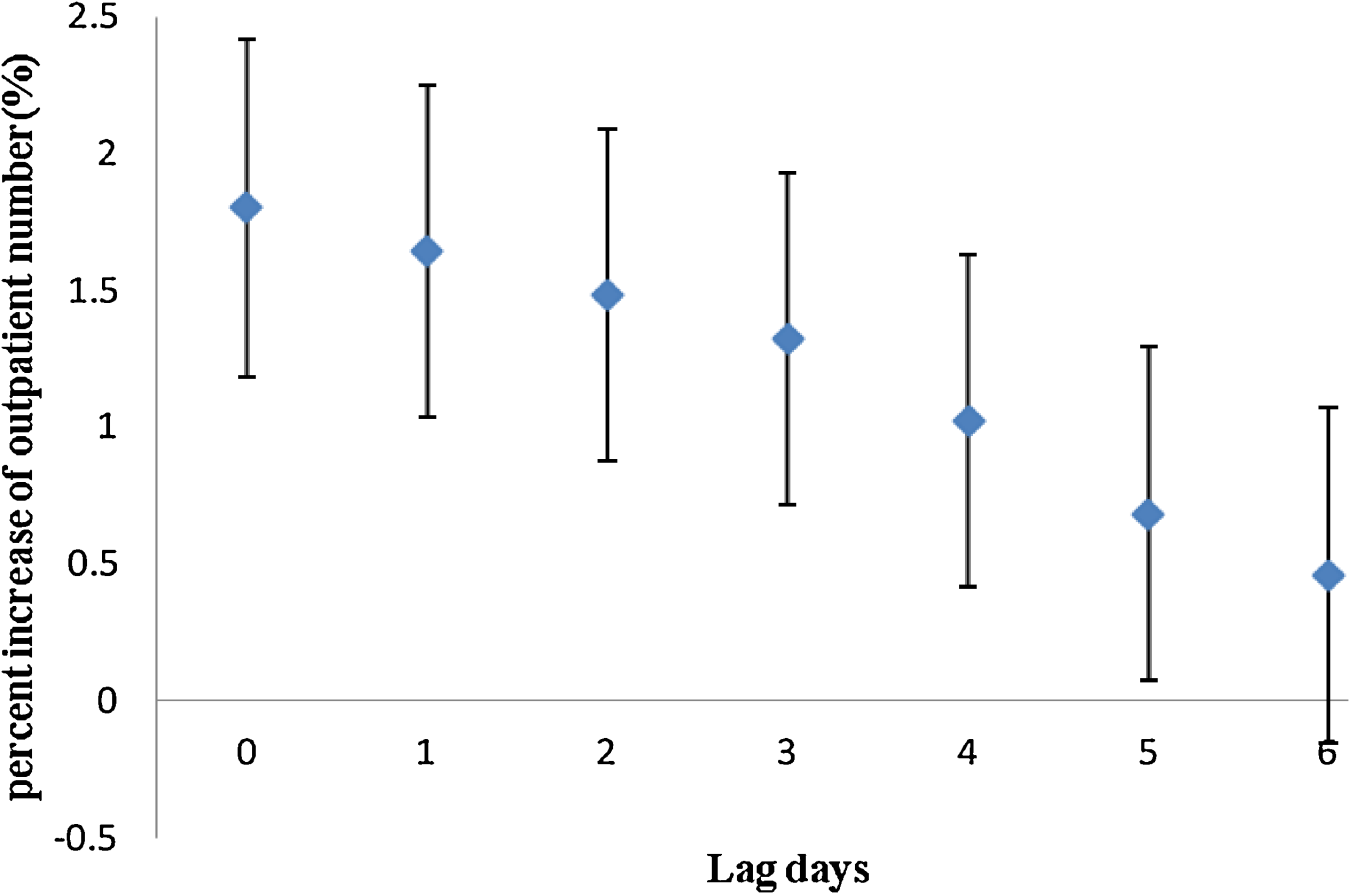
Percent increase in number of outpatient visits for diarrhea associated with a 1°C increase in mean temperature, by lag period.

As shown in Figure 2, mean temperatures on the current day and during the previous 5 days were positively associated with infectious diarrhea, although the effect was larger for the current day (lag 0). We found no significant association between rainfall and infectious diarrhea.

**Figure 3.**
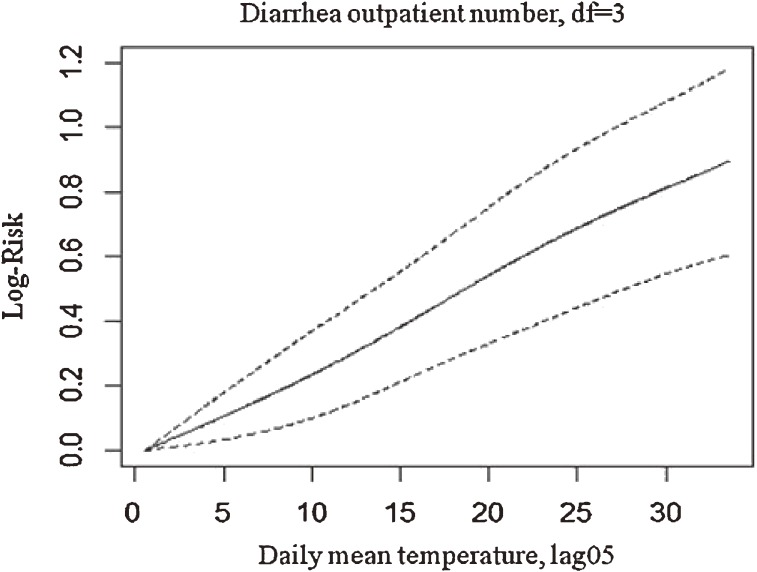
Exposure–response curve of mean temperature (lag 05) by number of outpatient visits for diarrhea. df, degrees of freedom.

In addition to the effect of single lag days, we also estimated the effect of the 6-day moving average of mean temperature (lag 05) on infectious diarrhea. A 1°C increase in the 6-day moving average of temperature was associated with a 2.68% (95% CI: 1.83%, 3.52%) increase in outpatient visits for diarrhea. Figure 3 shows the exposure–response curve, which indicates a linear relationship without an apparent threshold.

Sensitivity analysis showed that the effects of maximum and minimum temperatures on infectious diarrhea were similar to those of mean temperature, suggesting our results were robust with respect to the selection of the various temperature metrics (Table 4). Also, change in degrees of freedom per year for the time trend did not substantially affect the estimated effects of temperature (data not shown), indicating that our findings were relatively robust in this regard.

**Table 4.** Percent increase in outpatient visits for infectious diarrhea, by lag period, per 1°C increase in mean, maximum, and minimum temperature

Lag	Mean temperature		Maximum temperature		Minimum temperature	
		
	Increase %	95% CI	Increase %	95% CI	Increase %	95% CI
0	1.80	1.19, 2.42	1.42	0.94, 1.90	1.71	1.06, 2.36
1	1.64	1.03, 2.25	1.46	1.00, 1.92	1.31	0.67, 1.95
2	1.48	0.88, 2.09	1.35	0.89, 1.81	1.08	0.46, 1.70
3	1.32	0.72, 1.93	1.11	0.65, 1.58	0.99	0.37, 1.61
4	1.02	0.42, 1.63	1.10	0.63, 1.56	0.64	0.02, 1.27
5	0.68	0.07, 1.29	0.71	0.24, 1.18	0.24	−0.38, 0.86

## DISCUSSION

We found that high temperature was associated with increased risk of infectious diarrhea in Shanghai. To our knowledge, this is one of only a few studies in China that have examined the association between temperature and infectious diarrhea. The rapid increase in greenhouse gasses is expected to increase temperatures around the world. Our findings suggest that public health programs should focus on preventing diarrhea related to high temperature among urban residents.

Although the mechanism underlying the present association is unclear, there are several possible etiologic and meteorologic explanations for our findings. First, temperature may directly or indirectly influence the replication and survival of pathogens that cause diarrhea. For example, rotavirus and some bacteria that cause diarrhea proliferate in warm marine waters. Moe et al found that human rotavirus infectivity was lost more rapidly at 37°C than at 4°C or 20°C, regardless of humidity.^22^ In addition, vectors, such as plankton, that carry microbes proliferate faster in warm waters. Moreover, food poisoning, another important cause of diarrhea, occurs more often in warm weather because food spoils more easily when temperatures are higher.^23^ Second, studies suggest that diarrhea infectivity is closely related to temperature variation and region. A study of acute diarrhea among inpatients in northern Japan showed that human rotavirus increased abruptly when the mean temperature of any 10-day period became less than 5°C, reached a peak when it was less than 0°C, and waned when it became greater than 20°C.^2^ Third, dietary patterns and hygiene behavior might vary in relation to temperature. For example, demand for water is higher on hot days, which could facilitate transmission of bacteria and other pathogens.^24^

High temperature was associated with increased risk of infectious diarrhea. The effects of maximum and minimum temperatures were similar to that of mean temperature. Our findings were mostly consistent with those of previous studies. For example, daily admissions for diarrhea significantly increased among Peruvian children younger than 10 years during the El Niño–Southern Oscillation, and each 1°C increase in temperature was associated with an 8% increase in the risk of severe childhood diarrhea.^5^ In sub-Saharan Africa, the prevalence of diarrhea increased among children younger than 3 years as average monthly maximum temperature increased.^25^ A historical study in Shenyang that used classification and regression trees found that relative humidity, temperature, and atmospheric pressure 1 month earlier affected transmission of bacillary dysentery.^15^ In 2 studies, Zhang and colleagues found that a 1°C rise in maximum temperature was associated with an 11% increase in bacillary dysentery in Jinan City^16^ and a 16% increase in Baoan,^13^ and that a 1°C rise in minimum temperature was associated with a 12% increase in Jinan and a 14% increase in Baoan.^13^^,^^16^

Rainfall may affect levels of contaminants in drinking water, especially in places where fresh water is the main source of drinking water.^8^^–^^11^ In sub-Saharan Africa, a shortage of rainfall during the dry season increased diarrhea prevalence.^25^ A US study of outbreaks of waterborne disease from 1948 to 1994 found that 68% of outbreaks were preceded by precipitation events above the 80th percentile (*P* = 0.001) and that outbreaks caused by contamination of surface water had the strongest association with extreme precipitation.^26^ In our analysis, due to lack of precipitation data, rainfall was not included as a continuous variable but rather as a dummy variable; thus, we found no significant association between rainfall and infectious diarrhea.

Our study has several strengths. First, Shanghai is located in the middle of China and has a subtropical humid monsoon climate. Our study is the first in China to link temperature with infectious diarrhea in this region. Second, most previous studies examined specific causes of infectious diarrhea. For example, rotavirus diarrhea and gastroenteritis in children have been extensively studied in many countries.^27^^–^^35^ In China, however, the results of microbiologic examinations are complicated and confusing due to overuse of antibiotics. Therefore, our use of the general term “infectious diarrhea” in this study might be more useful from a public health perspective. Third, most prior studies considered hospital admissions or inpatients only; outpatients were not analyzed, which might have caused selective bias. To avoid this possibility, we collected data on numbers of outpatients and inpatients. Finally, most previous studies in China were conducted before 2003. Given the substantial changes since that time in the characteristics of diarrhea in China, studies based on the latest data are of great importance.

Our study has limitations. Due to the absence of data from microbiologic analysis, the illnesses of the present patients could not be classified by cause. In addition, infectious and noninfectious diarrhea may have different seasonal trends. Because data on noninfectious diarrhea were unavailable, such differences would not be revealed. In addition, the results could be influenced by unknown confounding factors, such as socioeconomic status.

In summary, high temperature was associated with increased risk of infectious diarrhea in Shanghai. Because temperature will continue to rise in the future, our findings may have important public health implications. The Chinese government should implement programs to prevent problems due to temperature-related diarrhea among urban residents.
